# 1,1′-[Imidazolidine-1,3-diylbis(methyl­ene)]bis­(1*H*-benzotriazole)

**DOI:** 10.1107/S1600536812000232

**Published:** 2012-01-11

**Authors:** Augusto Rivera, Diego Quiroga, Jaime Ríos-Motta, Karla Fejfarová, Michal Dušek

**Affiliations:** a Departamento de Química, Universidad Nacional de Colombia, Ciudad Universitaria, Bogotá, Colombia; b Institute of Physics ASCR, v.v.i., Na Slovance 2, 182 21 Praha 8, Czech Republic

## Abstract

In the title compound, C_17_H_18_N_8_, the imidazolidine ring adopts an envelope conformation with the substituents at the N atoms in *trans* positions with respect to the central ring. The dihedral angle between the two benzotriazole rings is 71.65 (10)°. In the crystal, non-classical C—H⋯N inter­actions link the mol­ecules into helical chains along the *b* axis. The crystal packing is further stabilized by weak C—H⋯π inter­actions.

## Related literature

For related structures, see: Rivera *et al.* (2011**a* ▶,b* ▶). For the synthesis of the title compound, see: Rivera *et al.* (2004 ▶); Katriztky *et al.* (1990 ▶). For ring conformations, see Cremer & Pople (1975 ▶). For bond-length data, see: Allen *et al.* (1987 ▶). For the anomeric effect, see: Dabbagh *et al.* (2002 ▶); Selámbaron *et al.* (2001 ▶); Zefirov & Shekhtman (1971 ▶); Hendrickson (1961 ▶).
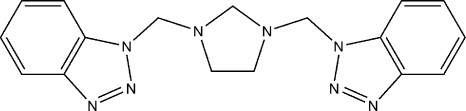


## Experimental

### 

#### Crystal data

C_17_H_18_N_8_
*M* *_r_* = 334.4
Monoclinic, 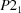

*a* = 11.8609 (6) Å
*b* = 4.6429 (2) Å
*c* = 14.4712 (8) Å
β = 93.053 (4)°
*V* = 795.78 (7) Å^3^
*Z* = 2
Cu *K*α radiation
μ = 0.74 mm^−1^
*T* = 120 K
0.43 × 0.18 × 0.10 mm

#### Data collection

Agilent Xcalibur diffractometer with an Atlas (Gemini ultra Cu) detector
Absorption correction: multi-scan (*CrysAlis PRO*; Agilent, 2010 ▶) *T*_min_ = 0.378, *T*_max_ = 1
10081 measured reflections
1609 independent reflections
1541 reflections with *I* > 3σ(*I*)
*R*_int_ = 0.030

#### Refinement

*R*[*F* ^2^ > 2σ(*F* ^2^)] = 0.027
*wR*(*F* ^2^) = 0.073
*S* = 1.52
1609 reflections
226 parameters
H-atom parameters constrained
Δρ_max_ = 0.09 e Å^−3^
Δρ_min_ = −0.11 e Å^−3^

### 

Data collection: *CrysAlis PRO* (Agilent, 2010 ▶); cell refinement: *CrysAlis PRO*; data reduction: *CrysAlis PRO*; program(s) used to solve structure: *SIR2002* (Burla *et al.*, 2003 ▶); program(s) used to refine structure: *JANA2006* (Petříček *et al.*, 2006 ▶); molecular graphics: *DIAMOND* (Brandenburg & Putz, 2005 ▶); software used to prepare material for publication: *JANA2006*.

## Supplementary Material

Crystal structure: contains datablock(s) global, I. DOI: 10.1107/S1600536812000232/bt5768sup1.cif

Structure factors: contains datablock(s) I. DOI: 10.1107/S1600536812000232/bt5768Isup2.hkl

Supplementary material file. DOI: 10.1107/S1600536812000232/bt5768Isup3.cml

Additional supplementary materials:  crystallographic information; 3D view; checkCIF report

## Figures and Tables

**Table 1:** Hydrogen-bond geometry (Å, °) *Cg*3 is the centroid of the N6/N7/N8/C13/C12 aromatic ring.

*D*—H⋯*A*	*D*—H	H⋯*A*	*D*⋯*A*	*D*—H⋯*A*
C17—H17⋯N5^i^	0.96	2.60	3.552 (2)	173
C11—H11*b*⋯*Cg*3^ii^	0.96	2.86	3.394 (2)	116

Symmetry codes: (i) 
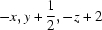
; (ii) 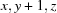.

**Fig. 1.**
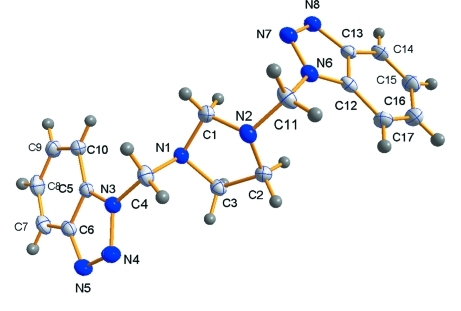
A view of (I) with the numbering scheme.displacement ellipsoids are drawn at the 50% probability level.

**Fig. 2.**
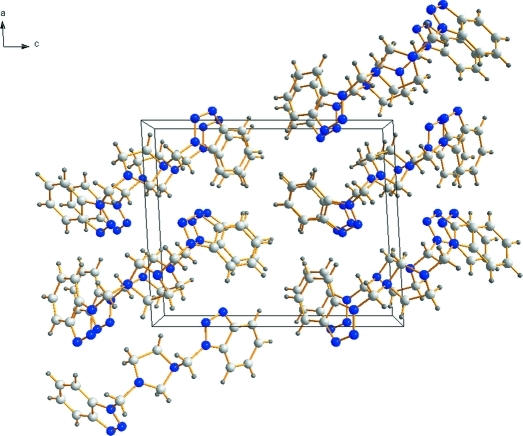
Packing of the molecules of the title compound view along b axis.

## supplementary crystallographic information

### Comment

The anomeric effect is a stereoelectronic effect observed in various heterocyclic compounds and some acyclic structures with a great deal of importance due its implications on the molecular structure, conformational properties and reactivity of organic compounds (Zefirov & Shekhtman, 1971; Selámbaron, *et al.*, 2001; Dabbagh, *et al.*, 2002). Our investigations on the synthesis and structural studies of heterocyclic compounds have evidenced the occurrence of a n(N)→σ* (C—N) electron delocalization (Rivera *et al.*, 2011*a*, 2011*b*). In this article, we discussed the crystal structure of the title compound, which can be synthetized by a three component condensation between ethylenediamine, formaldehyde and benzotriazole (Katriztky *et al.*, 1990) or using a novel methodology involving a Mannich type reaction between the aminal cage 1,3,6,8-tetrazatricyclo[4.4.1.1^3,8^]dodecane and benzotriazole (Rivera *et al.*, 2004). By recrystallization from ethanol we obtained suitable crystals for X-rays analysis.

The molecular structure and atom-numbering scheme for (I) are shown in Fig. 1. The anomeric effect is evidenced by the C—N bond lengths, which are longer as C11—N6 [1.484 (2) Å] and shorter as N2—C11 [1.433 (2) Å] than the expected bond length of 1.469 Å (Allen *et al.*, 1987). Moreover, this effect is confirmed by the bond angles around N2 with a Σα = 339.43 (13) which are distorted from a normal tetrahedral geometry in a five-membered ring (Hendrickson, 1961), whereas for N1 the bond lenghts and angles are within normal ranges. These results are in a good agreement with the crystal structures of related structures (Rivera *et al.*, 2011*a*, 2011*b*).

The imidazolidine ring adopts an envelope conformation on C1 as seen in the puckering parameters Q(2) = 0.3953 (17) Å and φ2 = 40.3 (2) ° (Cremer & Pople, 1975), with endocyclic bond angles between 103.06 (13) ° and 106.55 (13) °. The geometry of the N—C—C—N moiety is close to the planar in a *syn*-periplanar conformation evidenced by the N2—C2—C3—N1 torsion angle [3.05 (17) °]. The benzotriazolylmethyl substituents are arranged *trans* respect the imidazolidine ring, which is preferred because the nitrogen lone pairs are oriented anti-axial to avoid repulsion electronic repulsions. The benzotriazole rings makes an angle of 38.47 (10) ° and 78.88 (10) ° with the mean plane of imidazolidine ring. The dihedral angle between the two benzotriazole rings is 71.65 (10) °. Chains of molecules in the title compound are linked along the *b* direction by non-classical intermolecular hydrogen bonds C17—H17···N5 interactions [2.60 Å] which link neighboring molecules. The crystal packing is further stabilized by weak C—H···π interactions.

### Experimental

For the originally reported synthesis, see: Rivera *et al.* (2004). Single crystals of the title compound (I) were grown from ethanol by recrystallization.

### Refinement

All H atoms atoms were positioned geometrically and treated as riding on their parent atoms. The isotropic atomic displacement parameters of hydrogen atoms were evaluated as 1.2×*U*_eq_ of the parent atom. As the structure contains only light atoms, the Friedel-pair reflections were merged and the Flack parameter has not been determined.

### Crystal data

**Table:**

C_17_H_18_N_8_	*F*(000) = 352
*M**_r_* = 334.4	*D*_x_ = 1.395 Mg m^−^^3^
Monoclinic, *P*2_1_	Cu *K*α radiation, λ = 1.5418 Å
Hall symbol: P 2yb	Cell parameters from 7090 reflections
*a* = 11.8609 (6) Å	θ = 3.1–66.9°
*b* = 4.6429 (2) Å	µ = 0.74 mm^−^^1^
*c* = 14.4712 (8) Å	*T* = 120 K
β = 93.053 (4)°	Prism, colourless
*V* = 795.78 (7) Å^3^	0.43 × 0.18 × 0.10 mm
*Z* = 2	

### Data collection

**Table:**

Agilent Xcalibur diffractometer with an Atlas (Gemini ultra Cu) detector	1609 independent reflections
Radiation source: Enhance Ultra (Cu) X-ray Source	1541 reflections with *I* > 3σ(*I*)
mirror	*R*_int_ = 0.030
Detector resolution: 10.3784 pixels mm^-1^	θ_max_ = 67.0°, θ_min_ = 3.1°
Rotation method data acquisition using ω scans	*h* = −14→14
Absorption correction: multi-scan (CrysAlis PRO; Agilent, 2010)	*k* = −5→5
*T*_min_ = 0.378, *T*_max_ = 1	*l* = −17→16
10081 measured reflections	

### Refinement

**Table:**

Refinement on *F*^2^	73 constraints
*R*[*F*^2^ > 2σ(*F*^2^)] = 0.027	H-atom parameters constrained
*wR*(*F*^2^) = 0.073	Weighting scheme based on measured s.u.'s *w* = 1/(σ^2^(*I*) + 0.0016*I*^2^)
*S* = 1.52	(Δ/σ)_max_ = 0.005
1609 reflections	Δρ_max_ = 0.09 e Å^−^^3^
226 parameters	Δρ_min_ = −0.11 e Å^−^^3^
0 restraints	

### Special details

**Table:**

Experimental. CrysAlisPro (Agilent, 2010) Empirical absorption correction using spherical harmonics, implemented in SCALE3 ABSPACK scaling algorithm.
Refinement. The refinement was carried out against all reflections. The conventional *R*-factor is always based on *F*. The goodness of fit as well as the weighted *R*-factor are based on *F* and *F*^2^ for refinement carried out on *F* and *F*^2^, respectively. The threshold expression is used only for calculating *R*-factors *etc*. and it is not relevant to the choice of reflections for refinement.The program used for refinement, Jana2006, uses the weighting scheme based on the experimental expectations, see _refine_ls_weighting_details, that does not force *S* to be one. Therefore the values of *S* are usually larger than the ones from the *SHELX* program.

### Fractional atomic coordinates and isotropic or equivalent isotropic displacement parameters (Å^2^)

**Table:**

	*x*	*y*	*z*	*U*_iso_*/*U*_eq_	
N1	0.21591 (11)	0.2699 (3)	0.93068 (9)	0.0235 (4)	
N2	0.26794 (11)	0.5272 (3)	1.06164 (10)	0.0238 (4)	
N3	0.09989 (11)	0.3594 (3)	0.79499 (9)	0.0235 (4)	
N4	−0.01384 (12)	0.4004 (4)	0.78915 (11)	0.0290 (4)	
N5	−0.05795 (11)	0.2425 (4)	0.72197 (10)	0.0298 (5)	
N6	0.40908 (11)	0.4060 (3)	1.18741 (10)	0.0239 (4)	
N7	0.50606 (11)	0.2991 (4)	1.15561 (10)	0.0280 (4)	
N8	0.54874 (11)	0.1110 (4)	1.21481 (10)	0.0279 (4)	
C1	0.31519 (14)	0.3661 (4)	0.98560 (11)	0.0264 (5)	
C2	0.17294 (13)	0.3532 (4)	1.09029 (11)	0.0248 (5)	
C3	0.13569 (13)	0.1890 (4)	1.00110 (11)	0.0239 (5)	
C4	0.17205 (15)	0.4928 (4)	0.86759 (12)	0.0263 (5)	
C5	0.12961 (13)	0.1659 (4)	0.73030 (11)	0.0215 (5)	
C6	0.02769 (13)	0.0916 (4)	0.68333 (12)	0.0253 (5)	
C7	0.02635 (16)	−0.1107 (4)	0.61123 (12)	0.0314 (5)	
C8	0.12773 (17)	−0.2263 (5)	0.58977 (12)	0.0354 (6)	
C9	0.23000 (15)	−0.1486 (5)	0.63788 (12)	0.0308 (5)	
C10	0.23362 (14)	0.0484 (4)	0.70895 (11)	0.0251 (5)	
C11	0.34550 (15)	0.6304 (4)	1.13345 (12)	0.0286 (5)	
C12	0.38817 (13)	0.2843 (4)	1.27059 (11)	0.0228 (5)	
C13	0.47863 (13)	0.0943 (4)	1.28781 (12)	0.0232 (5)	
C14	0.48715 (14)	−0.0715 (4)	1.36880 (12)	0.0270 (5)	
C15	0.40331 (15)	−0.0378 (4)	1.42939 (13)	0.0311 (5)	
C16	0.31195 (15)	0.1530 (5)	1.41080 (13)	0.0329 (6)	
C17	0.30163 (14)	0.3172 (4)	1.33186 (12)	0.0293 (5)	
H1a	0.358632	0.493853	0.949314	0.0316*	
H1b	0.355874	0.201865	1.010035	0.0316*	
H2a	0.113022	0.477804	1.107635	0.0298*	
H2b	0.199161	0.21839	1.136922	0.0298*	
H3a	0.140331	−0.014517	1.012342	0.0287*	
H3b	0.060918	0.24844	0.980747	0.0287*	
H4a	0.128774	0.628658	0.901023	0.0316*	
H4b	0.233758	0.58913	0.840354	0.0316*	
H7	−0.042768	−0.165557	0.578371	0.0376*	
H8	0.129465	−0.364408	0.540509	0.0424*	
H9	0.299061	−0.236079	0.620438	0.037*	
H10	0.302981	0.101822	0.741735	0.0302*	
H11a	0.30616	0.75146	1.174811	0.0343*	
H11b	0.397607	0.762446	1.107664	0.0343*	
H14	0.54877	−0.2024	1.381195	0.0325*	
H15	0.406631	−0.146438	1.485921	0.0373*	
H16	0.254861	0.1683	1.45514	0.0395*	
H17	0.239268	0.445844	1.319532	0.0351*	

### Atomic displacement parameters (Å^2^)

**Table:**

	*U*^11^	*U*^22^	*U*^33^	*U*^12^	*U*^13^	*U*^23^
N1	0.0240 (6)	0.0259 (8)	0.0205 (7)	−0.0003 (6)	−0.0004 (5)	0.0012 (6)
N2	0.0261 (7)	0.0225 (7)	0.0223 (7)	−0.0021 (6)	−0.0037 (5)	0.0009 (6)
N3	0.0221 (6)	0.0253 (7)	0.0229 (7)	0.0011 (6)	−0.0008 (5)	0.0020 (6)
N4	0.0230 (7)	0.0315 (8)	0.0326 (8)	0.0042 (7)	0.0022 (6)	0.0053 (7)
N5	0.0218 (7)	0.0346 (9)	0.0325 (8)	−0.0010 (6)	−0.0027 (5)	0.0075 (7)
N6	0.0235 (6)	0.0241 (8)	0.0235 (7)	−0.0008 (6)	−0.0032 (5)	−0.0013 (6)
N7	0.0227 (6)	0.0338 (9)	0.0269 (7)	−0.0032 (6)	−0.0025 (5)	−0.0021 (7)
N8	0.0221 (7)	0.0336 (9)	0.0275 (7)	0.0006 (6)	−0.0023 (5)	−0.0019 (7)
C1	0.0233 (7)	0.0334 (10)	0.0224 (8)	−0.0017 (8)	0.0009 (6)	0.0024 (8)
C2	0.0212 (7)	0.0296 (9)	0.0236 (8)	0.0009 (7)	0.0008 (6)	−0.0008 (7)
C3	0.0233 (8)	0.0239 (9)	0.0243 (8)	−0.0025 (7)	−0.0007 (6)	0.0003 (7)
C4	0.0323 (8)	0.0244 (9)	0.0219 (8)	−0.0026 (8)	−0.0032 (6)	−0.0010 (7)
C5	0.0234 (7)	0.0221 (9)	0.0187 (7)	−0.0006 (7)	−0.0008 (6)	0.0035 (7)
C6	0.0240 (8)	0.0260 (9)	0.0255 (8)	−0.0022 (7)	−0.0033 (6)	0.0080 (7)
C7	0.0360 (9)	0.0301 (10)	0.0267 (9)	−0.0058 (8)	−0.0098 (7)	0.0031 (8)
C8	0.0514 (11)	0.0314 (10)	0.0228 (8)	−0.0009 (9)	−0.0021 (7)	−0.0015 (8)
C9	0.0332 (8)	0.0330 (11)	0.0264 (8)	0.0063 (8)	0.0038 (6)	0.0011 (8)
C10	0.0229 (8)	0.0291 (10)	0.0235 (8)	−0.0004 (7)	0.0009 (6)	0.0029 (7)
C11	0.0348 (9)	0.0208 (9)	0.0288 (9)	−0.0039 (8)	−0.0109 (7)	0.0012 (8)
C12	0.0224 (7)	0.0225 (9)	0.0228 (8)	−0.0029 (7)	−0.0062 (6)	−0.0028 (7)
C13	0.0193 (7)	0.0246 (9)	0.0253 (8)	−0.0022 (7)	−0.0032 (6)	−0.0039 (7)
C14	0.0262 (8)	0.0241 (9)	0.0299 (9)	0.0014 (7)	−0.0066 (6)	0.0000 (7)
C15	0.0322 (9)	0.0317 (11)	0.0289 (9)	−0.0030 (8)	−0.0028 (7)	0.0043 (8)
C16	0.0292 (8)	0.0394 (11)	0.0303 (9)	−0.0009 (8)	0.0039 (7)	−0.0004 (9)
C17	0.0238 (8)	0.0336 (11)	0.0302 (9)	0.0041 (8)	−0.0013 (6)	−0.0026 (8)

### Geometric parameters (Å, °)

**Table:**

N1—C1	1.455 (2)	C4—H4b	0.96
N1—C3	1.479 (2)	C5—C6	1.398 (2)
N1—C4	1.458 (2)	C5—C10	1.398 (2)
N2—C1	1.466 (2)	C6—C7	1.403 (3)
N2—C2	1.464 (2)	C7—C8	1.368 (3)
N2—C11	1.433 (2)	C7—H7	0.96
N3—N4	1.3604 (19)	C8—C9	1.413 (3)
N3—C4	1.458 (2)	C8—H8	0.96
N3—C5	1.357 (2)	C9—C10	1.376 (3)
N4—N5	1.305 (2)	C9—H9	0.96
N5—C6	1.377 (2)	C10—H10	0.96
N6—N7	1.355 (2)	C11—H11a	0.96
N6—C11	1.484 (2)	C11—H11b	0.96
N6—C12	1.364 (2)	C12—C13	1.401 (2)
N7—N8	1.307 (2)	C12—C17	1.400 (2)
N8—C13	1.381 (2)	C13—C14	1.402 (2)
C1—H1a	0.96	C14—C15	1.369 (3)
C1—H1b	0.96	C14—H14	0.96
C2—C3	1.543 (2)	C15—C16	1.414 (3)
C2—H2a	0.96	C15—H15	0.96
C2—H2b	0.96	C16—C17	1.373 (3)
C3—H3a	0.96	C16—H16	0.96
C3—H3b	0.96	C17—H17	0.96
C4—H4a	0.96		
			
C1—N1—C3	103.48 (12)	N3—C5—C10	132.45 (15)
C1—N1—C4	112.04 (14)	C6—C5—C10	123.13 (15)
C3—N1—C4	112.98 (13)	N5—C6—C5	108.33 (15)
C1—N2—C2	105.19 (14)	N5—C6—C7	131.54 (15)
C1—N2—C11	117.30 (13)	C5—C6—C7	120.12 (16)
C2—N2—C11	116.94 (13)	C6—C7—C8	117.14 (16)
N4—N3—C4	121.83 (14)	C6—C7—H7	121.4291
N4—N3—C5	110.05 (13)	C8—C7—H7	121.4287
C4—N3—C5	127.90 (14)	C7—C8—C9	122.02 (18)
N3—N4—N5	108.94 (14)	C7—C8—H8	118.9882
N4—N5—C6	108.26 (13)	C9—C8—H8	118.9897
N7—N6—C11	119.68 (14)	C8—C9—C10	122.01 (17)
N7—N6—C12	110.21 (14)	C8—C9—H9	118.9967
C11—N6—C12	130.08 (14)	C10—C9—H9	118.9971
N6—N7—N8	109.17 (13)	C5—C10—C9	115.58 (15)
N7—N8—C13	108.11 (14)	C5—C10—H10	122.2124
N1—C1—N2	103.65 (13)	C9—C10—H10	122.2126
N1—C1—H1a	109.4707	N2—C11—N6	115.81 (15)
N1—C1—H1b	109.4711	N2—C11—H11a	109.4718
N2—C1—H1a	109.4712	N2—C11—H11b	109.4704
N2—C1—H1b	109.4715	N6—C11—H11a	109.4709
H1a—C1—H1b	114.7236	N6—C11—H11b	109.4712
N2—C2—C3	103.06 (13)	H11a—C11—H11b	102.2888
N2—C2—H2a	109.4714	N6—C12—C13	104.13 (14)
N2—C2—H2b	109.4715	N6—C12—C17	133.44 (16)
C3—C2—H2a	109.4717	C13—C12—C17	122.43 (16)
C3—C2—H2b	109.4711	N8—C13—C12	108.38 (15)
H2a—C2—H2b	115.2	N8—C13—C14	130.61 (16)
N1—C3—C2	106.55 (13)	C12—C13—C14	121.01 (15)
N1—C3—H3a	109.4715	C13—C14—C15	116.76 (16)
N1—C3—H3b	109.4715	C13—C14—H14	121.6205
C2—C3—H3a	109.4712	C15—C14—H14	121.6212
C2—C3—H3b	109.4712	C14—C15—C16	121.66 (17)
H3a—C3—H3b	112.2427	C14—C15—H15	119.1684
N1—C4—N3	109.01 (15)	C16—C15—H15	119.1674
N1—C4—H4a	109.4712	C15—C16—C17	122.64 (17)
N1—C4—H4b	109.4703	C15—C16—H16	118.6785
N3—C4—H4a	109.4717	C17—C16—H16	118.6776
N3—C4—H4b	109.4721	C12—C17—C16	115.49 (16)
H4a—C4—H4b	109.9289	C12—C17—H17	122.2546
N3—C5—C6	104.42 (14)	C16—C17—H17	122.2552
			
N2—C2—C3—N1	3.05 (17)		

### Hydrogen-bond geometry (Å, °)

**Table:**

*Cg*3 is the centroid of the N6/N7/N8/C13/C12 aromatic ring.

**Table:**

*D*—H···*A*	*D*—H	H···*A*	*D*···*A*	*D*—H···*A*
C17—H17···N5^i^	0.96	2.60	3.552 (2)	173
C11—H11b···Cg3^ii^	0.96	2.86	3.394 (2)	116

Symmetry codes: (i) −*x*, *y*+1/2, −*z*+2; (ii) *x*, *y*+1, *z*.
